# Intra-fractional geometric and dose/volume metric variations of magnetic resonance imaging-guided stereotactic radiotherapy of prostate bed after radical prostatectomy

**DOI:** 10.1016/j.phro.2024.100573

**Published:** 2024-03-23

**Authors:** Yu Gao, Stephanie Yoon, Ting Martin Ma, Yingli Yang, Ke Sheng, Daniel A. Low, Leslie Ballas, Michael L. Steinberg, Amar U Kishan, Minsong Cao

**Affiliations:** a Department of Radiation Oncology, University of California, Los Angeles, Los Angeles, CA, USA; b Department of Radiation Oncology, Stanford University, Palo Alto, CA, USA; c Department of Radiation Oncology, City of Hope, Duarte, CA, USA; d Department of Radiation Oncology, University of Washington, Seattle, WA, USA; e Department of Radiation Oncology, Shanghai Ruijin Hospital, China; f Department of Radiation Oncology, University of California, San Francisco, San Francisco, CA, USA; g Department of Radiation Oncology, Cedars-Sinai Medical Center, Los Angeles, CA, USA

**Keywords:** Post-prostatectomy, Prostate cancer, MRI guided radiotherapy, MRgRT, Intrafractional variations

## Abstract

**Background and purpose:**

Magnetic Resonance Imaging (MRI)-guided Stereotactic body radiotherapy (SBRT) treatment to prostate bed after radical prostatectomy has garnered growing interests. The aim of this study is to evaluate intra-fractional anatomic and dose/volume metric variations for patients receiving this treatment.

**Materials and methods:**

Nineteen patients who received 30–34 Gy in 5 fractions on a 0.35T MR-Linac were included. Pre- and post-treatment MRIs were acquired for each fraction (total of 75 fractions). The Clinical Target Volume (CTV), bladder, rectum, and rectal wall were contoured on all images. Volumetric changes, Hausdorff distance, Mean Distance to Agreement (MDA), and Dice similarity coefficient (DSC) for each structure were calculated. Median value and Interquartile range (IQR) were recorded. Changes in target coverage and Organ at Risk (OAR) constraints were compared and evaluated using Wilcoxon rank sum tests at a significant level of 0.05.

**Results:**

Bladder had the largest volumetric changes, with a median volume increase of 48.9 % (IQR 28.9–76.8 %) and a median MDA of 5.1 mm (IQR 3.4–7.1 mm). Intra-fractional CTV volume remained stable with a median volume change of 1.2 % (0.0–4.8 %). DSC was 0.97 (IQR 0.94–0.99). For the dose/volume metrics, there were no statistically significant changes observed except for an increase in bladder hotspot and a decrease of bladder V_32.5 Gy_ and mean dose. The CTV V_95%_ changed from 99.9 % (IQR 98.8–100 %) to 99.6 % (IQR 93.9–100 %).

**Conclusion:**

Despite intra-fractional variations of OARs, CTV coverage remained stable during MRI-guided SBRT treatments for the prostate bed.

## Highlights

• Median bladder volume increase during treatment was 48.8%
• Clinical Target Volume size and dose remained stable between pre- and post- treatment scans.
• No significant intra-fractional organ at risk dose changes were observed for the rectum.
• Post-treatment bladder had slightly higher hotspot compared to pre-treatment.

## 1 Introduction

Postoperative radiation therapy (RT) has shown to be an effective treatment for prostate cancer patients with biochemical recurrence following radical prostatectomy [1], [2], [3], [4], [5]. However, postoperative RT has not been well adopted, due to the prolonged treatment course of the conventional fractionated RT, physician biases, and concerns for toxicity [6], [7]. Hypofractionated treatment has the advantage of a shorter treatment course to facilitate the adoption of postoperative RT. Early studies using 51.0–52.5 Gy in 17–20 fractions have shown comparable disease control outcomes without significant late toxicity compared to conventionally fractionated RT [8], [9]. There has been a growing interest in adopting ultra-hypo fractionated stereotactic body radiotherapy (SBRT) using 30–40 Gy in five fractions for post-prostatectomy with promising results [10], [11], [12]. Nevertheless, randomized trials are still needed to investigate the role of hypofractionation and SBRT for postoperative treatment [13], [14].

In contrast to the intact prostate, where the target definition and intra-fraction motion has been well-studied [15], [16], [17], [18], a primary challenge in prostate bed treatment arises from the highly variable nature of the clinical target volume (CTV) that depends on the location and shape of the bladder and rectum. Large inter-fraction changes and deformation of the rectum and bladder make accurate target alignment challenging [19], [20], [21], [22]. There have been several studies investigating inter- and intra- fractional motion for conventional fractionated treatment using ultrasound, a real-time electromagnetic transponder system, location of surgical clips on Cone-beam computed tomography systems (CBCT), or soft-tissue match between pre- and post-CBCT [23], [24], [25], [26], [27], [28]. Small translational volume motions, ranging from submillimeter to over 2 mm, were observed in these studies. In a study with patients receiving CBCT-guided SBRT to the prostate bed [29], the impact of intra-fractional motion on dose/volume metrics was evaluated and acceptable CTV coverage was met in about 70 % of patients. However, CBCT exhibits low soft-tissue contrast and is susceptible to imaging artifacts, potentially limiting the precision of assessments.

Magnetic Resonance Imaging (MRI)-guided radiation therapy (MRgRT) offers the advantages of superior soft-tissue contrast for target delineation, enhanced soft-tissue based treatment setup, and real-time target tracking and gating, thus enabling tighter planning target volume (PTV) margins [30]. In the context of intact prostate cancer, a randomized trial demonstrated that aggressive margin reduction can lead to reduced toxicity [31]. For prostate cancer after radical prostatectomy, preliminary findings have indicated a reduction in gastrointestinal (GI) toxicity when treated with MRgRT using a 3 mm PTV margin [32]. However, it is necessary to validate that adequate target coverage can be achieved with this tighter margin concerning inter- and intra-fraction motion. An analysis of inter-fractional variations revealed mild changes in prostate bed coverage and OAR constraint violations in almost half of the treatment sessions, which could be improved through adaptive planning [33].

While inter-fractional motion could be effectively accounted for by couch correction and adaptive planning, intra-fractional motion remains a concern, especially in MRgRT treatments. These treatments tend to be longer than Computed Tomography (CT)-guided treatment due to a lower dose rate and the step-and-shoot delivery technique. The objective of this study was to investigate intra-fractional anatomic and dose/volume metrics variations in patients receiving MRI-guided SBRT treatment following radical prostatectomy with a tight 3 mm margin. To the best of our knowledge, this is the first study evaluating intra-fractional dosimetry changes for prostate bed SBRT treatment on an MRI-guided linear accelerator (MR-Linac).

## 2 Materials and methods

### 2.1 Patient treatment scheme

This retrospective study involved patients who had undergone post-prostatectomy radiotherapy and participated in a phase II clinical trial (NCT03541850). This trial aimed to investigate the toxicity, quality of life, and treatment efficacy of SBRT to the prostate bed [32], and patients received treatment on either regular Linac or a 0.35T MRI-guided Linac (MRIdian, ViewRay Inc. Cleveland, OH, USA). Patients of this study were retrospectively selected from the MR-Linac treatment group receiving a dose of 34 Gy in 5 fractions every other day. Lower target dose coverage in the range of 30–34 Gy was allowed to prioritize OAR sparing. Organs at risks (OARs) and prostate bed CTV were delineated based on the MR simulation scan, with the target volumes defined in accordance with the Radiation Therapy Oncology Group (RTOG) consensus guideline [34]. The PTV was generated with a 3 mm isotropic expansion to the CTV, as opposed to a 5 mm margin in the regular Linac group. The rationale for the reduced PTV margin was the enhanced soft-tissue contrast provided by MRI, enabling improved setup accuracy and reduced setup uncertainty, as well as the gating capability to minimize target motion during treatment. For patients with visible gross tumor, a gross tumor volume was defined and expanded isotopically by 3 mm to form a PTV_Gross_. A simultaneously integrated boost of 40 Gy was prescribed to the PTV_Gross_, along with the treatment to the prostate bed. Additionally, one patient received elective pelvic nodal radiation of 25 Gy, as decided by the treating physician.

### 2.2 Simulation and treatment planning

Patients were instructed to follow institutional bladder and rectum preparation protocol for the MR simulation as well as the subsequent treatment. The preparation includes taking an enema the night before and the morning of the appointment to empty the rectum, voiding the bladder one hour prior to the appointment, and then drinking 470–710 ml of water to maintain a comfortably full bladder during the appointment. During the radiation planning simulation, an MR scan was acquired using the 0.35T on-board MR scanner. The image was acquired using a 172-second balanced steady-state free precession (bSSFP) sequence with 1.5 mm isotropic resolution and 50 × 45 × 43 cm^3^ field of view. A CT scan was acquired on the same day as the MR simulation on a Siemens SOMATOM Definition AS CT using 120 kVp, 500 mAs, 0.97 mm in-plane resolution and 1.5 mm slice thickness. The same immobilization device was employed for the CT and MRI simulations. During treatment planning, this CT scan was deformably registered to the planning MR scan to obtain electron density information for dose calculation.

The treatment plans were optimized using the ViewRay MRIdian treatment planning system, which employed a Monte Carlo-based algorithm to account for the magnetic field effect on dose distribution. Intensity-modulated radiation therapy (IMRT) plans with 10–19 beams were generated at 2 mm dose calculation resolution for each patient. Radiation treatment planning objectives included: PTV V_100%_ > 95 %; bladder maximum dose (D_max_) < 35.7 Gy (relaxed to 39 Gy for patients with PTV_Gross_), V_32.5Gy_ < 35 %, Rectum D_max_ < 35.7 Gy (relaxed to 39 Gy for plans with PTV_Gross_), V_32.5Gy_ < 30 %, V_27.5Gy_ < 45 %, and rectal wall V_24Gy_ < 50 %. OAR dose constraints were prioritized over PTV coverage during plan optimization. The constraints were developed based on institutional experience and constraints used in other clinical trials [10].

### 2.3 Intra-fractional imaging and data collection

Before the start of each treatment fraction, an MR scan (pre-treatment MRI), using the same bSSFP sequence as in the simulation, was acquired to assist with patient setup. Rigid registration was performed to align the pre-treatment MRI and simulation MRI scans, focusing on the bladder and rectum interface. This fusion was reviewed by a physicist and a physician before applying the corresponding couch correction. During the treatment, Cine MRI at 4 frames per second were acquired to monitor patient movement. A portion of the anterior rectal wall was selected as the gating structure. If the detected gating structure moved out of a pre-specified margin, treatment was held automatically until the gating structure moved into the margin. After radiation treatment, another bSSFP scan (post-treatment MRI) was acquired to assess intra-fractional anatomical changes.

A total of 31 patients were enrolled in the MRI-guided group for this clinical trial. However, some patients opted out the optional post-treatment MRI due to discomfort or unwillingness to hold the bladder at the end of treatment session. Machine faults led to interruptions in 15 treatment fractions, resulting in partial radiation plan delivery before the interruptions. During these interruptions, some patients opted to partially empty their bladder. To ensure a clean dataset for consistent intra-fractional analysis, we retained only those patients with a minimum of 2 fractions containing complete and uninterrupted data. A total of 75 fractions from 19 patients were included in this study. Each patient had 2–5 fractions (median of 4 fractions) included.

### 2.4 Data evaluation and statistical analysis

The total treatment time for each patient was recorded, which was determined as the time interval between post-treatment MRI and pre-treatment MRI, encompassing daily image alignment, physician and physicist review, as well as radiation treatment delivery. Patient setup was not included in the reported treatment time.

To evaluate intra-fractional anatomical changes, a single radiation oncologist retrospectively contoured prostate bed CTV, rectum, rectal wall, and bladder on fractional pre-treatment and post-treatment MRIs for all 19 patients. Contours were reviewed by the principal investigator. Metrics including percent volume changes, absolute volume changes, Hausdorff distance (HD), Mean Distance to Agreement (MDA), and Dice similarity coefficient (DSC) were calculated for each structure to quantify the intra-fractional anatomical variations.

To investigate the impact of anatomical changes on dose/volume metrics, the baseline radiation dose from pre-treatment scans was transferred to post-treatment MRI. Differences of calculated dose/volume metrics between the pre- and post-treatment scans were then quantified, where CTV coverage, OAR plan objectives, and mean doses to both CTV and OARs were assessed.

For each evaluated variable, the median value and interquartile range (IQR) were recorded. Wilcoxon rank sum tests were utilized to determine the statistical significance of dosimetry metric changes at a significant level of 0.05. To examine potential correlations between treatment time and volumetric changes, Pearson correlation coefficients (R) were calculated between treatment time and volumetric changes. Furthermore, Pearson correlation coefficients were computed between changes in dose/volume metrics and changes in the corresponding structure’s anatomical metrics (percent volume change, absolute volume change, and DSC). Interpretation of the correlation coefficient was based on Mukaka [35].

## 3 Results

The median total treatment time of the analyzed 75 fractions was 29 min (IQR 24–33 min).

Structural anatomical changes were summarized in Table 1. The bladder experienced the largest changes with a median volume increase of 48.9 % (IQR 28.9–76.8 %). The median HD between pre-treatment and post-treatment bladder was 20.9 mm (IQR 15.8–28.6 mm), and median MDA was 5.1 mm (3.4–7.1 mm). In contrast, CTV volume remained relatively stable with a median volume change of 1.2 % (IQR 0.0–4.8 %), and a high DSC of 0.97 (IQR 0.94–0.99). The rectum had a smaller median volume change compared to the CTV, but its DSC was lower (median DSC 0.85, IQR 0.79–0.88). Regarding the rectal wall, the median DSC was 0.51 (IQR 0.41–0.59), indicating variation in shape or position.

**Table 1:** Intrafractional anatomy changes quantified as percent volume change, absolute volume change, HD, MDA, and DSC.

		CTV	Bladder	Rectum	Rectal wall
Percent volume change (%)	Median	1.2	48.9	1.1	−0.1
	Q1	0.0	28.9	−6.2	−4.2
	Q3	4.8	76.8	8.5	4.5

Absolute volume change (cm^3^)	Median	1.4	140.5	0.5	0.0
	Q1	0.0	72.8	−3.7	−1.2
	Q3	4.9	180.9	4.8	1.2

HD (mm)	Median	5.5	20.9	9.1	8.9
	Q1	4.5	15.8	6.7	6.5
	Q3	7.7	28.6	11.4	11.1

MDA (mm)	Median	0.4	5.1	1.5	1.1
	Q1	0.2	3.4	1.2	0.9
	Q3	0.9	7.1	2.1	1.4

DSC	Median	0.97	0.79	0.85	0.51
	Q1	0.94	0.72	0.79	0.41
	Q3	0.99	0.86	0.88	0.59

Calculated dose/volume metrics based on pre-treatment and post-treatment MRI were listed in Table 2. Overall, there was a slight decrease in the median CTV V_100%_ coverage from 98.3 % (IQR 93.5–99.5 %) to 96.8 % (IQR 88.7–99.1 %). Median CTV V_95%_ remained stable, but there was a larger variation post-treatment (IQR 93.9 %-100.0 %) compared to pre-treatment (IQR 98.8–100.0 %). Based on post-treatment MRI, 5 fractions had CTV coverage lower than the acceptable constraint V_95%_ <93 % due to the intrafraction motion. CTV mean dose remained stable. Significant differences were observed in all metrics for the bladder between pre- and post-treatment scans. Metrics indicated hot spots worsened: the median V_35.7Gy_ increased from 0 cm^3^ (IQR 0.0–0.1 cm^3^) to 0.1 cm^3^ (IQR 0.0–1.6 cm^3^), and the maximum point dose increased from 35.7 Gy (IQR 35.4–37.1 Gy) to 36.9 Gy (IQR 35.6–38.7 Gy). Mean bladder dose and V_32.5Gy_ decreased favorably. In the case of the rectum and rectal wall, the median values of all metrics either remained similar or decreased between pre- and post-treatment MRI, though these changes were not statistically significant.

**Table 2:** Dosimetry metrics based on pre-treatment MRI and post-treatment MRI.

Dosimetry metrics	Pre-treatment			Post-treatment			p-value
	median	Q1	Q3	median	Q1	Q3	
CTV V_100%_ (%)	98.3	93.5	99.5	96.8	88.7	99.1	0.19
CTV V_95%_ (%)	99.9	98.8	100.0	99.6	93.9	100.0	0.09
CTV mean dose (Gy)	33.6	32.9	34.6	33.6	32.8	34.6	0.56
Bladder V_35.7Gy_ (cm^3^)*	0.0	0.0	0.1	0.1	0.0	1.6	**0.00**
Bladder V_32.5Gy_ (%)*	18.7	11.6	28.4	13.3	9.5	19.7	**0.01**
Bladder D_max_ (Gy)*	35.7	35.4	37.1	36.9	35.6	38.7	**0.02**
Bladder mean dose (Gy)*	17.5	12.4	22.5	12.6	9.9	19.1	**0.00**
Rectum V_35.7Gy_ (cm^3^)	0.0	0.0	0.0	0.0	0.0	0.0	0.99
Rectum V_32.5Gy_ (%)	0.7	0.2	3.0	0.5	0.0	3.3	0.59
Rectum V_27.5Gy_ (%)	7.1	3.5	13.5	5.1	1.7	13.2	0.18
Rectum D_max_ (Gy)	34.6	33.7	36.2	34.2	33.2	36.2	0.22
Rectum mean dose (Gy)	15.6	12.8	17.9	15.2	12.7	17.9	0.72
Rectal wall V_24Gy_ (%)	18.2	11.5	25.4	14.9	7.9	25.4	0.24
Rectal wall mean dose (Gy)	16.2	13.2	18.1	15.7	13.1	18.4	0.74

(* p < 0.05 between dosimetric parameter based on pre-treatment MRI and post-treatment MRI).

**Fig. 1:**
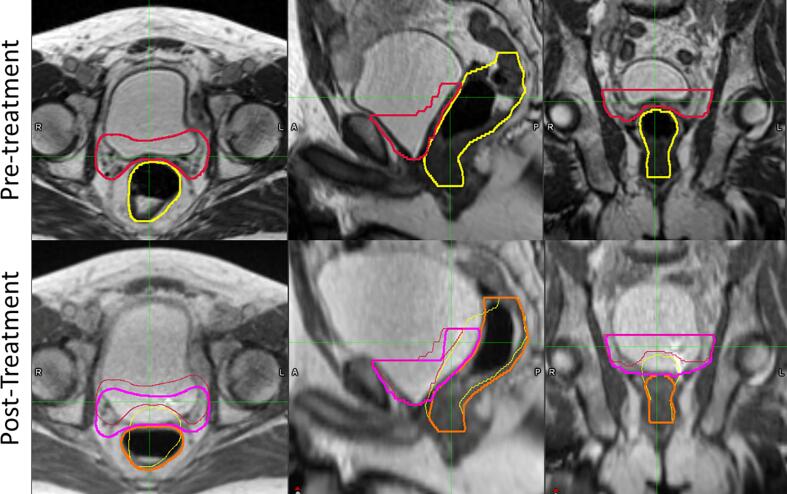
Visual comparison of pre-treatment and post-treatment MRIs for one patient. Pre-treatment CTV and rectum contours were displayed in red and yellow. Post-treatment CTV and rectum were displayed in magenta and orange.

Fig. 1 showed an example case with large anatomical changes during treatment due to bladder filling and gas in the rectum (treatment time 39 min). Due to the large anatomical changes, CTV V_95%_ coverage decreased from 96.7 % pre-treatment to 81.8 % post-treatment, and bladder V_35.7Gy_ increased from 0.8 cm^3^ to 1.9 cm^3^. Rectal dose became more favorable, and the maximum dose was reduced from 34.7 Gy to 29.8 Gy.

Regarding correlation between treatment time and structural volumetric changes, low correlation was found for all structures. Specifically, the Pearson correlation coefficient between treatment time and bladder percent/absolute volume change was all 0.23.

In terms of the correlation between intra-fractional dose/volume metrics changes and volume/DSC changes, Pearson correlation coefficients were summarized in Table 3. In general, percentage volume changes exhibited higher correlations with dose/volume metrices changes than either absolute volume changes or DSC. Among the 14 dose/volume metrics, percent volume changes demonstrated moderate correlation for 9 dose/volume metrics and low correlation with 5 metrics.

**Table 3:** Pearson correlation coefficient table showing the relationship between Δ dose metrics (post-treatment dose metrics minus pre-treatment dose metrics) and changes in the corresponding structure’s anatomical metrics (percent volume change, absolute volume change, and DSC). (Moderate correlation (0.5 to 0.7 or −0.7 to −0.5) was highlighted in bold.)

	Structure Percent volume change	Structure Absolute volume change	DSC
ΔCTV V_100%_	**−0.68**	**−0.58**	0.39
ΔCTV V_95%_	**−0.68**	**−0.55**	0.43
ΔCTV mean dose	**−0.61**	−0.47	0.40
ΔBladder V_35.7Gy_	**0.63**	0.39	−0.49
ΔBladder V_32.5Gy_	**−0.61**	−0.51	**0.61**
ΔBladder D_max_	**0.50**	0.23	0.48
ΔBladder mean dose	**−0.57**	−0.37	**0.64**
ΔRectum V_35.7Gy_	**0.60**	**0.67**	−0.20
ΔRectum V_32.5Gy_	**0.60**	**0.60**	−0.38
ΔRectum V_27.5Gy_	0.37	0.46	−0.27
ΔRectum maximum dose	0.26	0.28	0.17
ΔRectum mean dose	0.40	0.44	−0.09
ΔRectal wall V_24Gy_	0.42	0.40	0.09
ΔRectal wall mean dose	0.11	0.08	0.03

## 4 Discussion

In this study, we investigated the intra-fractional anatomical changes during SBRT treatment to the prostate bed and assessed the resultant changes in dose/volume metrics based on the patient anatomy obtained from pre-treatment and post-treatment MRI. Compared to conventional Linac SBRT, MR-Linac has much long treatment time, during which anatomical changes may be non-trivial. Hence, validating intra-fractional dosimetry is crucial to justify SBRT for the prostate bed on the MR-Linac system.

In this study, large bladder volume increase was observed due to continuous bladder filling during treatment, while the rectum and rectal wall had minor volume changes and varying degrees of deformation. Those changes were expected as a result of our bladder and rectum preparation protocol. Despite the anatomical changes, the target volume and shape remained relatively stable. As a result, no statistically significant changes affecting the target coverage were identified. In terms of the OAR dosimetry, slight increases were observed in the bladder V_35.7Gy_ and maximum point dose. This can be attributed to bladder filling, which caused distention of the posterior bladder wall into the planned high dose region. The increase in bladder size during treatment also resulted in a decrease of bladder V_32.5Gy_ and mean dose. Notably, no statistically significant changes were detected for the rectum and rectal wall despite shape changes. This aligns with the observation that most rectal distension led to expansion of the posterior rectal wall, which is located in the low dose bath region, while the bladder rectal interface remained relatively stable during the treatment.

In a related study from the same clinical trial, geometric and dose/volume metrics variations were assessed by comparing CBCT scans taken before and after treatment [29]. This study observed a similar decline in CTV coverage and a favorable decrease in the mean doses to the bladder and rectum. However, in our research, we noted a substantially greater change in bladder (median 48.9 % vs. 16.8 %), likely due to the longer treatment time on the MR-Linac (median of 29 min vs. 15.5 min between pre- and post-imaging).

It is worth noting that treatment beam gating, enabled by cine MR imaging, was utilized during treatment delivery for the patients in this study. Real-time beam gating can potentially minimize the impact of transient motion during treatment and identify large steady motion for appropriate intervention. The ability to track and gate the beam delivery in real time is a crucial factor, along with improved setup accuracy, that allowed a tight PTV margin in this study and likely contributed to the favorable toxicity profile observed in this trial [32]. Long-term follow-up is underway to assess late toxicity and the treatment outcome. Furthermore, it is important to acknowledge that inter-factional anatomic changes can be substantial [33], [36]. The benefits of adaptive MRI-guided SBRT will be studied prospectively in another clinical trial to take full utilization of MR guidance.

There are some limitations of the study. Firstly, the sample size of patients was relatively small. However, it is important to note that the 75 fractions included in the study encompassed a wide range of treatment times, varying from less than 20 min to over 40 min, and patients exhibited diverse intra-fractional bladder size changes. Despite the modest sample size, we believe that the results provide a representative understanding of inter-fractional anatomical changes in postprostatectomy SBRT treatment on the MR-Linac system for our specific bladder and rectum preparation protocol. Secondly, the dosimetry analysis in this study was based on pre- and post-treatment MRI, which might not accurately reflect the actual dose delivered. We did not account for the impact of transient movements during treatment, although beam gating largely mitigates the latter as shown in a recent study [37]. One should be cautious in interpreting the findings of this study which does not directly apply to conventional treatment without active beam gating. In addition, although sufficient CTV coverage was observed for majority of the treatment fractions, a small percentage of the fractions still exhibit suboptimal coverage, suggesting that a patient specific margin or tighter intra-fractional motion gating control might need to be employed for patients with large intra-fraction motion. Thirdly, although five different geometric metrics were analyzed to assess the anatomical changes, each metric comes with its own limitations and is not capable of fully representing spatial changes of the structures. Therefore, dosimetry evaluation is essential and performed in this study. Moreover, the results presented in this study were susceptible to inherent intra-observer and inter-observer contour variability [38]. To mitigate this, a single radiation oncologist contoured the CTV and OARs, and these contours were subsequently reviewed by the principal investigator overseeing the clinical trial. The use of MRI with its superior soft tissue contrast facilitated better delineation of the bladder and rectum, reducing ambiguity in the delineation process. Lastly, contouring in this study followed the conventional RTOG consensus. Prostate-Specific Membrane Antigen-Positron Emission Tomography (PSMA-PET) image guided target definition is still evolving [39] and the adequacy of margins and the integration of PSMA-PET data into clinical decision-making were not evaluated in this study which certainly warrants future investigation.

In conclusion, this retrospective study analyzing intra-fractional motion demonstrates that CTV dose coverage remains stable during treatment, despite changes in the shape and size of the bladder, rectum, and rectal wall. No significant intra-fractional OAR dose changes were observed for the rectum and rectal wall. Bladder experienced a significant increase in the hotspot and a reduction in both bladder V_32.5Gy_ and mean dose.

## CRediT authorship contribution statement

**Yu Gao:** Methodology, Formal analysis, Data curation, Writing – original draft. **Stephanie Yoon:** Data curation, Writing – review & editing. **Ting Martin Ma:** Data curation, Writing – review & editing. **Yingli Yang:** Resources. **Ke Sheng:** Resources, Writing – review & editing. **Daniel A. Low:** Resources. **Leslie Ballas:** Resources. **Michael L. Steinberg:** Resources. **Amar U Kishan:** Conceptualization, Resources, Writing – review & editing, Supervision. **Minsong Cao:** Conceptualization, Methodology, Formal analysis, Data curation, Writing – original draft.

## Declaration of competing interest

The authors declare the following financial interests/personal relationships which may be considered as potential competing interests: Dr. Kishan reports consulting fees and speaking honoraria from Varian Medical Systems, ViewRay, and Intelligent Automation; equity in ViewRay; and serving on the Janssen advisory board and Boston Scientific; received research funds from Janssen and Point Biopharma and ViewRay. Dr. Low reports corporate grant with Varian, consulting and presentation travel and fees from ViewRay, SAB for TAE Life Sciences, and founder of Pulmonum LLC. Dr. Ma and Dr. Steinberg reports honorarium from ViewRay. Dr. Cao reports corporate grant with Varian, honorarium and consulting fees from Varian and ViewRay. All other authors do not have any potential conflicts of interest to disclose. Funding support for the SCIMITAR trial was partially provided by ViewRay, Inc., but ViewRay, Inc. has no role in the conduct of this analysis.
